# Voyages in Vacation.—VII

**Published:** 1888-11-10

**Authors:** 


### VOYAGES IN VACATION.—VII.

By One in Search of Restful Restoratives.

(*Continued from page 77.*)

### COPENHAGEN AND THE BALTIC.

Having considered the various matters which it is necessary for those who desire to go a voyage for pleasure should know, it may prove interesting to complete this series of articles by a brief description of the Baltic cruise, apart from the ship and its doings. In the first article we described the journey to Copenhagen, where we arrived one Sunday morning, and at once visited the English church, a modern building with a shapely spire of red sandstone. We afterwards made a call upon a friend, and had the pleasure of seeing the Dane at home. An old-fashioned house, with all the roominess and comfort of an English country mansion, with a garden, too, of fair proportions—although our friend resided in a crowded street where such luxuries were least expected. Internally the house was furnished in excellent taste, some of the furniture being exquisitely carved, and so old and shapely as to make the eye of a connoisseur glisten with pleasure. Old engravings, an elegant, and, to English ideas, a terrible stove in each sitting-room; some pictures by eminent Danish artists, a quantity of birds quaintly arranged, in unexpected places, a library containing most of the best English, American, and foreign books of note, decorations in such good taste as to be remarkable, and a quiet homely comfort pervading everywhere, combined to make this bachelor's den—for he was a bachelor—one of the most enviable of pleasant resting-places. So snug and tasteful a home promoted peaceful contentment, and made one regret that so cheery an Eden was still unshared by an Eve. Our celibate drove thoughts of matrimony to the winds, and promptly stifled any erratic or heretical notions on the subject which contact with the outside world in Denmark might momentarily excite.

We found that the breakfast hour is usually eleven a.m., dinner being taken at half-past four, and supper after eight p.m. At breakfast three kinds of wine, port, sherry, and claret, are first served with some substantial dish like beef cutlets, then salad, after which an excellent and wholly novel soufflée which was apportioned out by the use of a sort of large fish knife. Tea and coffee followed, and then cheese, butter, and biscuits. Finally a white brandy with fruit is handed to each guest, followed by cigars and cigarettes. Of course, if one has commenced the day early and done half-a-day's work before breakfast, such a substantial meal is very welcome at eleven. otherwise it is a little embarrassing to most novices. In Denmark, too, sandwiches have to be studied before being ordered, or the result may be anything but satisfactory. They are usually made of a size about two inches by four, on each piece of bread a thick layer of butter is first placed, and then between the two layers of bread, cheese, raw smoked goose (rather tasty), various kinds of raw and smoked fish, reindeer's tongue, nearly raw veal, a mixture of piccalilli, gerkins, or other tit bits are placed. Some of these sandwiches are excellent, but some do not commend themselves either to the eye or taste of the Englishman. At the hotels frequented by visitors, ordinary dishes are of course served, and the wine is reasonable and good.

Of Copenhagen itself, it may be said that it is prettily situated, that it has excellent shops, and one of the most famous porcelain manufactories in the world, but the paving of the streets and the drainage arrangements leave much to be desired. The people are frank, intelligent, and courteous. The ladies most attractive in appearance in manner and dress, but none of the lower orders wear bonnets. The humanity of the inhabitants is shown in a marked degree by the thoughtful care displayed towards their horses. Thus every cab stand has a number of crotchets upon which the horses rest their nose-bags when feeding. The French Museum and the Rosenberg Castle are particularly worthy of a visit. The exhibition was proceeding at the time of our arrival, and the feature which struck us most was the extraordinary badness of some of the pictures exhibited in all seriousness by Swedish and Norwegian artists (?). The great feature of Copenhagen is undoubtedly the Tivoli Gardens, which on a Sunday evening present an appearance so attractive and novel as to be surprising. Every kind of amusement is provided, from skittles to the drama, and from common swings to balloon ascents. The most extraordinary contrivance was a circular platform on which boats were placed, which was so arranged that when set going the boats pitched and rolled as the platform revolved with a variety and uncomfortableness of motion which far surpassed the most unpleasant experiences of sea transit. There were quite 30,000 people at the Tivoli the night we were there, all of whom were enjoying themselves in a quiet, orderly way, the chief attraction being a series of concerts in the open air and within the Kursaal, which was crowded with interested and intelligent listeners. The behaviour and habits of the people as here seen make a deep impression upon all foreigners, who cannot fail to mark and appreciate the intelligence and culture which are apparent to an astonishing degree amongst all classes of the population of Copenhagen.

As we left Copenhagen we were again struck by the number of vessels encountered, pointing as it did to a brisk trade with foreign countries. Our experience of the Baltic was altogether pleasant. The sea was on the whole smooth, and even when a squall was encountered it was soon apparent that the seas here resemble those of the North Sea. There are nearly always, in rough weather, two short waves followed by a long one which often strikes a vessel with astonishing violence. A large steamer generally takes the two short waves and about half of the succeeding one, so that water is not unfrequently shipped in consequence. Of course, there is nothing so grand as "an Atlantic roller" to be seen in a storm either in the Baltic or North Seas, though the character of the waves gives a peculiarly unpleasant motion to the steamer which usually proves fatal to most passengers after a brief experience. The roll of the *Victoria* may be gathered from the following description of the motion of a circular disc cast upon the wall of the cabin by the sunlight passing through a porthole. A stroke of from three to one and a-half feet upwards and downwards, caused by "the swell of the ocean," then half a turn on the ship's axis to the left—prolonged and extended to three-quarters of a turn sometimes—accompanied by a nerve-shaking whirligig at the termination of each stroke. Next came a straight up-and-down movement, then the half or three-quarter turn—to the right this time with the whirligig aforesaid—with a most trying back twist thrown in. All these motions were present in a modified or extended degree according to the state of the sea. The curious reader, who has unlimited time and patience, ought to be able to work out with pencil and paper a fair picture of the movements we experienced; especially if a circle of about three inches in diameter is first drawn in the centre of the paper. How bravely some fought against the deadly influence of these most upsetting gyrations of screw and sea, whilst others, alas, all too soon collapsed, must not be told, or we fear actions for slander and defamation might prove more numerous than pleasant should the editor overlook the paragraphs in question. Such little matters, however, are but trifles after all, mere "showers in summer" to the cheerful voyageur, who is ever buoyed up with hope by the thoughts of land and other pleasures, that he knows are sure to come all in good time.

(*To be continued.*)

